# Spatiotemporal changes in riverine input into the Eocene North Sea revealed by strontium isotope and barium analysis of bivalve shells

**DOI:** 10.1038/s41598-024-79779-0

**Published:** 2024-11-20

**Authors:** Jorit F. Kniest, David Evans, Axel Gerdes, Marjorie Cantine, Jonathan A. Todd, Julia D. Sigwart, Johan Vellekoop, Wolfgang Müller, Silke Voigt, Jacek Raddatz

**Affiliations:** 1 Institute of Geosciences, Goethe University Frankfurt, Frankfurt, Germany; 2 Frankfurt Isotope and Element Research Center (FIERCE), Goethe University Frankfurt, Frankfurt, Germany; 3 School of Ocean & Earth Science, University of Southampton, Southampton, UK; 4 Department of Earth and Space Sciences, University of Washington, Seattle, USA; 5 The Natural History Museum, London, UK; 6 Department of Marine Zoology, Senckenberg Institute and Natural History Museum, Frankfurt, Germany; 7 Department of Earth and Environmental Sciences, KU Leuven, Leuven, Belgium; 8 Operational Directorate Earth and History of Life, Institute of Natural Sciences, Brussels, Belgium; 9 Present Address: GEOMAR Helmholtz Centre for Ocean Research Kiel, Kiel, Germany

**Keywords:** Biogeochemistry, Climate sciences, Environmental sciences, Hydrology, Ocean sciences

## Abstract

Reconstructing hydrological conditions of past warm periods, such as the Eocene ‘hot house’ provides empirical data to compare to state of the art climate models. However, reconstructing these changes in deep time is challenging, for example, given the complex interplay between evapotranspiration, precipitation and runoff. As a proxy for past changes in these hydrological systems, the dynamics of fresh water input into marginal seas can be used to identify the spatiotemporal distribution of riverine runoff. Elemental barium (Ba) and radiogenic strontium (^87^Sr) are, depending on the amount of runoff and the background geology of the catchment area, typically enriched in river waters in comparison to seawater and can thus be utilized to determine changes in riverine fresh water discharge. Here, we use barium to calcium ratios (Ba/Ca) and radiogenic strontium isotopes (^87^Sr/^86^Sr) measured in fossil bivalve shells to reconstruct patterns of fresh water input into the paleo North Sea during the early to middle Eocene. Our reconstruction shows the potential of Ba/Ca and ^87^Sr/^86^Sr to serve as proxies for riverine runoff and highlights the spatiotemporal complexity of Eocene hydrological conditions in western Europe. In particular, our results enable changes in riverine input along geological to perennial time scales for different coastal regions to be determined, revealing a steady influx of fresh water, but with distinct spatiotemporal differences.

**Supplementary Information:**

The online version contains supplementary material available at 10.1038/s41598-024-79779-0.

## Introduction

Understanding the details of the hydrological cycle in warm periods in Earth’s geological past is key knowledge in understanding climate system dynamics and is useful empirical information which may be compared to climate simulations in order to ultimately improve predictions of future climate change^1^. In particular, the Eocene has been the target of numerous investigations as an informative interval to study ‘high CO_2_’ climate states, since this period experienced the warmest global temperatures during the Cenozoic^2–4^. However, developing a complete understanding of hydrological patterns in the geological past is challenging, due to the complex interplay and spatial patterns of evapotranspiration, precipitation and runoff^1^. To address this issue, an examination of the individual components of the hydrological system is needed to deduce reliable climate information.

Marginal seas can record freshwater runoff through riverine input, as these shallow marine areas are an intersection between the continents and the open ocean. The radiogenic isotope of strontium (^87^Sr) and barium are typically present in river waters at a relatively high concentration due to the weathering of bedrock material in the catchment area of rivers^5–7^. The radiogenic strontium (^87^Sr/^86^Sr) and the barium to calcium ratio (Ba/Ca) in fossil carbonate shells can therefore be employed as proxies to identify the influence of freshwater input and accompanied salinity changes^8–11^. Fossil mollusc shells are an ideal archive to track such ^87^Sr/^86^Sr and Ba/Ca variability in coastal waters across a range of spatiotemporal resolutions due to the time-discrete (tidal to annual) layer structure of their shells (growth increments)^12^ and their widespread abundance and temporal continuity in the geological record. Due to their quasi-sessile mode of life, they are able to record local variations in the barium to calcium ratio and strontium isotopic composition of seawater on geological time to sub-annual scales. Additionally, some bivalves can tolerate considerable changes in salinity through freshwater input. This is true, for example, of selected members of the order Carditida, including the genera *Venericor* and *Crassatella*, which thrived in very shallow coastal areas to open marine environments^13–17^ and which are utilized here.

**Fig. 1:**
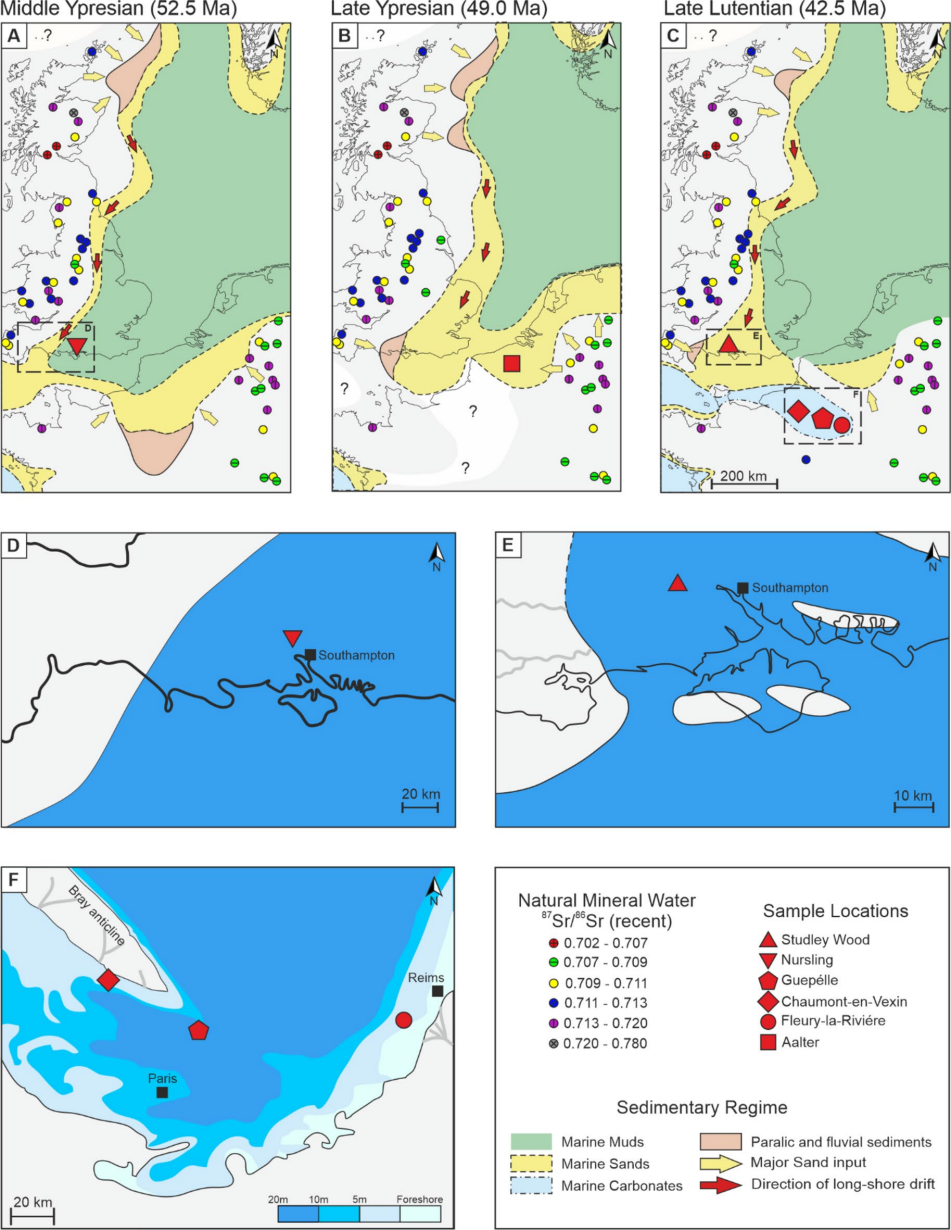
Palaeogeography of the Eocene North Sea basins with the ^87^Sr/^86^Sr of recent natural mineral waters overlain. (**A–C**) Evolution of the paleogeography and sedimentary regime of the paleo North Sea from the early to middle Eocene (edited after Knox et al.^18^). The ^87^Sr/^86^Sr of recent natural mineral waters is shown by the coloured symbols (edited after Voerkelius et al.^19^). (**D**,**E**) Reconstruction of the paleo coast line and sedimentary regime for the Hampshire basin (edited after Knox et al.^18^ and Gale et al.^20^). (**F**) Reconstruction of the paleo coast line and water depth of the Paris Basin (edited after Gely and Merle^21^ and Sanders et al.^22^).

In this study, we use laser ablation inductively-coupled-plasma mass-spectrometry to measure sub-annual resolved ^87^Sr/^86^Sr and Ba/Ca in thirteen pristinely preserved fossil bivalve shells (see “Materials and methods”) from the southern paleo North Sea (Fig. 1; Table 1). The proxy data is used to reconstruct riverine runoff signals in three contiguous marginal sea basins (Paris Basin, Hampshire Basin, Belgian Basin) during the early to middle Eocene (53 to 40 Ma). Using this approach we show changes in hydrological patterns on different spatiotemporal scales from regional to local, as well as along geological periods to perennial intervals, gaining further insight into Eocene hydrological variability.

## Results

Thirteen individual bivalve shells originating from three separate basins along the southern margin of the paleo North Sea were analysed for this study. The three basins comprise several sample sites, detailed in Table 1. A more comprehensive overview of the age and stratigraphy for the different sample sites is given in supplementary data 3. Specimens from all locations are characterized by aragonitic preservation of the shell material (Supplementary Fig. S1d).

**Table 1:** Overview of sampling localities and age of the bivalve specimens utilized here.

Basin	Age range	Sample Site	Stratigraphic unit	Species (number of specimens)
Hampshire	40.5–41.5	Studley Wood	Huntingbridge Shell Bed, Elmore Member^18^	*Venericor planicosta* (2)
	52.5–52.9	Nursling	Nursling Member^16^	Venericor sp. nov. (2)
Paris	43.4–44.0	Chaumont-en-Vexin	Calcaire grossier moyen^19^	*Venericor planicosta* (3)
	44.0–45.0	Fleury-La-Rivière	Calcaire grossier moyen^19^	*Crassatella ponderosa* (3)
	39.3–41.03	Le Guépelle	Sables du Guépelle^19^	*Venericor planicosta* (1)
Belgian	47.5–49.0	Aalter	Oedelem Sand Member^20^	*Venericor planicosta lerichei* (2)

### Strontium isotopic heterogeneity

**Fig. 2:**
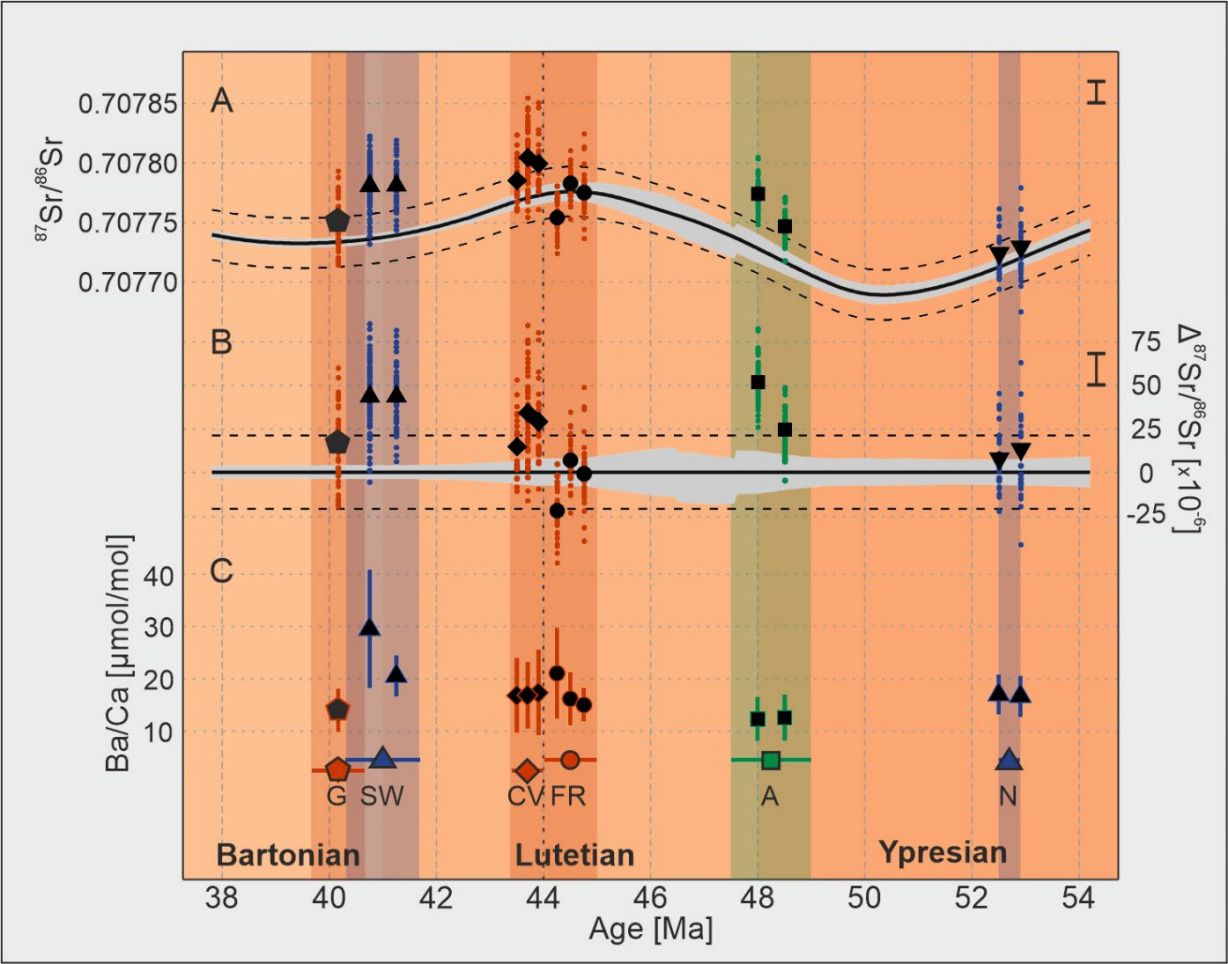
Strontium isotopic composition and barium baseline values of the fossil bivalve shells. (**A**) mean values (black symbols) and single measurements (coloured points) of ^87^Sr/^86^Sr from bivalve shells; the Eocene global seawater ^87^Sr/^86^Sr reconstruction is from McArthur et al.^23^ (black curve, grey shading—uncertainties of the LOWESS fit) with modern oceanic water ^87^Sr/^86^Sr variability (± 21 × 10^−6^, dashed lines)^6^; average standard error for single measurements is displayed by the black vertical bar (2 SE = 18 × 10^−6^); (**B**) mean values (black symbols) and single measurement (coloured points) of ^87^Sr/^86^Sr from bivalve shells normalised to the relevant Eocene seawater ^87^Sr/^86^Sr value^23^; the seawater ^87^Sr/^86^Sr value for normalisation is that for the mean age of each sampling site (Supplementary Material 2); average standard error for single measurements is displayed a black vertical bar (2 SE = 18 × 10^−6^); (**C**) bivalve shell Ba/Ca baseline (± 2SD); coloured symbols indicate mean age and horizontal bars, while the shaded areas show the age uncertainty for each sampling location, in which the plotted samples may fall (Table 1); *G* Le Guépelle, *SW* Studley Wood, *CV* Chaumont-en-Vexin, *FR* Fleury-La-Rivière, *A* Aalter, *N* Nursling; basins are indicated by colour: Paris Basin — red, Hampshire Basin — blue, Belgian Basin — green. Eocene stages are shown in the same colours as the geological time scale^24^.

Mean ^87^Sr/^86^Sr shell values derived using laser ablation multi-collector inductively-coupled-plasma mass-spectrometry range from 0.707724 to 0.707804. The range of mean values from different specimens collected from the same sample site varies from less than 1.0 × 10^−6^ (far lower than analytical reproducibility) for the two specimens from Studley Wood up to 27.3 × 10^−6^ for the shells from Aalter. All specimens are characterised by internal variability beyond that which can be explained by the precision of the analytical technique, ranging from 53.4 to 104.2 × 10^−6^. In order to compare these values to the corresponding global ^87^Sr/^86^Sr seawater value, we use the data compilation of McArthur et al.^23^ to which we add an envelope of ± 21 × 10^−6^ which is the modern coastal seawater variability^6^ (Fig. 2a, b). Almost all specimens analysed here intersect with this 21 × 10^−6^ interval within their intra-shell ^87^Sr/^86^Sr variability, although the degree to which this heterogeneity overlaps with the temporally-equivalent open ocean values varies both within and among sample sites. In general, these fossil shells tend to more radiogenic values relative to the global seawater record, repeatedly exceeding the typical modern coastal seawater variability envelope. Exceptions to this are the samples from Nursling and Fleury-La-Rivière, which are characterised by mean ^87^Sr/^86^Sr within uncertainty of global seawater or in one case are less radiogenic (Fig. 2a, b). One important result from this dataset is the observation that the internal variation of ^87^Sr/^86^Sr isotopic values within a single shell can be larger than observed variation within modern coastal waters^6^ or known temporal variation across much of the Eocene^23^.

### Ba/Ca baseline

All analysed shells exhibit a similar pattern in terms of their Ba/Ca profiles in that they are characterised by a low and generally invariant baseline ratio (Ba/Ca_BL_) punctuated by recurring peaks (Supplementary Fig. S1b), which exceed the background by a factor of up to 10 to 45 (Supplementary Data 1). On average, peak heights range between 100 and 300 µmol/mol with some rare exceptions reaching values > 500 µmol/mol. The determined Ba/Ca_BL_ values (s. “Methods”) for each of the examined shells range from 12.3 to 29.5 µmol/mol with an average variability of 5.8 µmol/mol (2σ of all Ba/Ca_BL_ values within a specimen, Fig. 2c). With two exceptions (see below), mean baseline values between individual specimens from the same sample site plot within a narrow range relative to the magnitude of intra-individual Ba/Ca heterogeneity, differing by less than 1.0 µmol/mol. Exceptions to this are the specimens from Fleury-La-Rivière and from Studley Wood, differing by 6.0 µmol/mol and 9.0 µmol/mol, respectively (Supplementary Fig. S1c).

## Discussion

### Strontium isotopes as proxy for riverine input

The radiogenic strontium isotopic composition of aragonitic bivalve shells reflects ambient water ^87^Sr/^86^Sr ^6,25^. The incorporation of radiogenic strontium does not dependend on species or ontogeny^26^. This is also evident in the recent specimens analysed here (Supplementary Data 1).

In coastal areas changes in the radiogenic strontium isotope ratio of seawater are predominately influenced by riverine input^7^. However, submarine groundwater incursion can also potentially influence coastal waters, although the rate of discharge is considerably lower and typically characterised by an isotopic composition more similar to seawater than riverine input^27^. Therefore, the magnitude of the isotopic deviation of coastal seawater from the global mean mainly depends on the rate of riverine runoff and the ^87^Sr/^86^Sr value of the river water. In general, the ^87^Sr/^86^Sr signal of river water is primarily determined by the background geology of the catchment area via the effect that this has on the strontium isotopic composition of the bedrock and the ease with which it is leached during weathering. However, the isotopic composition of the river water can be seasonally variable depending on the rates of runoff, thus the seasonal distribution and amount of precipitation in the catchment area. These annual hydrological variations can result in seasonal changes in the ^87^Sr/^86^Sr signal of several 1000 × 10^−6^ at the river mouth^28,29^. However, the high concentration of Sr in seawater compared to most rivers means that the variability of the river water isotopic endmember is readily dampened, typically to less than a few 100 × 10^−6^ along the mouth area of the river, depending on the coastal regime^6^. As a result, the mean strontium isotopic composition of riverine runoff is temporally more constant on geological time scales and rather predominantly shifts with changes in the geology and geomorphology of the hinterland. However, there is currently no data set that allows for the reconstruction and description of such variations in the strontium isotopic composition of seawater along different spatiotemporal resolutions for paleo coastal areas.

Nonetheless, the different factors influencing the radiogenic strontium isotopic composition of coastal seawater on different spatiotemporal scales are evident in the ^87^Sr/^86^Sr dataset presented here. On an inter-regional scale, i.e. considering the entire southern paleo North Sea, the strontium isotopic composition of the analysed shells mainly follows the global ^87^Sr/^86^Sr seawater curve of the early to middle Eocene^23^ (Fig. 2a). Assessing the three marginal basins studied here individually reveals a regionally more heterogeneous distribution in ^87^Sr/^86^Sr between and within the contiguous areas in terms of the inter/intra-specimen variability and deviation from the global mean values of the analysed specimens (Fig. 2b). In this context, the observed changes in strontium isotopic composition correlate with both the paleo-oceanographic evolution of the basins as well as the ^87^Sr/^86^Sr ratio of the hinterland geology (Fig. 1). Here, this latter control is assessed using the ^87^Sr/^86^Sr values of recent mineral waters originating from pre-Eocene rock units in order to approximate the strontium isotopic composition of riverine runoff during the Eocene^19^. This approach is independent from the strontium concentration of the exposed basement rock, since only the soluble portion of the strontium can affect the radiogenic signal of the runoff. However, we acknowledge that uncertainties concerning the riverine input signal remain, since only limited data regarding catchment size exists while there are also large local variations in the isotopic composition of the mineral waters in some regions. Nonetheless, catchment relevant mineral water springs exceed the corresponding Eocene seawater ^87^Sr/^86^Sr value by several 1000 × 10^−6^ (Supplementary data 2), such that substantial riverine input is expected to be identifiable via strontium isotope analysis of the bivalves utilised here.

Specifically, large differences of the radiogenic strontium composition between riverine runoff and seawater are present in the Belgian Basin, where the freshwater signal mainly derives from weathering of Palaeozoic rocks of the Ardennes and thereby could potentially have exceeded that of seawater by several 10,000 × 10^−6^. This high ^87^Sr/^86^Sr value is also observed in the two specimens from Aalter, which are shifted to more radiogenic values, falling substantially above the confidence interval of the global seawater curve and projected coastal variability.

The hinterland geology of the Hampshire Basin was also likely characterised by a more radiogenic strontium isotopic composition, similar to the Belgian Basin^19^. This is because the weathering of late Palaeozoic rocks of southern Wales and south-western England, including the Dartmoor granite, which has a especially radiogenic strontium isotope composition^30,31^. However, of the four shells from the Hampshire Basin studied here, only the two late Lutetian specimens (Studley Wood) are characterised by an increased radiogenic signal, despite the likely radiogenic ^87^Sr/^86^Sr composition of the freshwater endmember. We hypothesise that this more complex pattern observed in the isotopic composition of the shells is related to the paleo-geographic and -oceanographic development of the Hampshire Basin from the early to middle Eocene. During the Ypresian (specifically, during the termination of sequence C1 of the London Clay) the specimen from Nursling thrived in an open marine environment, an offshore very shallow-water sandbar running parallel to the coastline, where any freshwater influence was presumably strongly dampened (Fig. 1d)^30,32–34^. In contrast, towards the end of the Lutetian (when the basal Elmore Member was deposited), specimens from Studley Wood lived above the storm wave base in fully marine transgressive sandy muds. At this time, the region had a more complex paleo-geography with a major river system supplying fresh water from nearby land directly to the west^20,30,33^ (Fig. 1e).

In contrast to the Belgian and Hampshire Basins, the background geology of the Paris Basin during the Eocene was mainly determined by Cretaceous and Jurassic sediments with a low radiogenic strontium isotopic composition, which are characterised by an ^87^Sr/^86^Sr ratio more similar to or even below that of Eocene seawater^19,35,36^. In accordance with this, the specimens of the Paris Basin exhibit the lowest ^87^Sr/^86^Sr values of all analysed shells. Within the basin, the ^87^Sr/^86^Sr values systematically vary between the three sampling sites, with the lowest ^87^Sr/^86^Sr ratios observed in the eastern part of the basin (at Fleury-La-Rivière) and increasing in a western direction to Le Guépelle and Chaumont-en-Vexin. This spatial pattern in Δ^87^Sr/^86^Sr most likely relates to different catchment areas contributing freshwater to the western and eastern parts of the basin. In the eastern part of the basin near Fleury-La-Rivière, riverine input most likely sampled Cretaceous sediments with ^87^Sr/^86^Sr values lower than Eocene seawater, whereas in the west at Chaumont-en-Vexin the freshwater runoff signal may have been influenced by the elevation of the Bray anticline^21,22^ (Fig. 1f). The Bray anticline also mainly consists of Cretaceous and Jurassic rocks^35,37^. However, as indicated by the ^87^Sr/^86^Sr values of the bivalve shells, the strontium isotopic composition was presumably more radiogenic than the eastern part of the basin. In addition, we stress that the Paris Basin was not a static environment, but underwent considerable changes in the extent of marine to brackish regimes within the ~ 4 Ma time span represented by the three examined sampling sites^38^, which limits our ability to estimate rates of runoff across the entire basin within a given time interval.

While the broad regional differences in the deviation of the ^87^Sr/^86^Sr values of the shells from global seawater can be largely attributed to the control of background geology on the likely composition of the fresh water flux, the local nuances in the pattern and short-term (intra-specimen) variations rather represent changes in catchment expansion and seasonally variable rate of runoff. The specimens from each sample site (except Studley Wood) show local variations in mean ^87^Sr/^86^Sr values. This short-term variability, in terms of geological time intervals, could result from millennial-scale movements of the catchment area, taking into account the likely amount of time averaging within the beds sampled at each site, which typically encompass several hundred thousand years in many or all cases. In addition, at the highest spatiotemporal resolution, the observed inter-shell ^87^Sr/^86^Sr heterogeneities, ranging from 53.4 to 104.2 × 10^−6^, likely reflects seasonal to perennial changes in the rate of runoff, since these are too large to result from analytical noise alone, which is on average 18 × 10^−6^ for each individual strontium isotope measurement (2× standard error; Fig. 2, Supplementary Fig. S1, Supplementary Data 1).

The intra-shell heterogeneity of the strontium isotopic composition of the shells analysed here can be used to estimate local salinity changes for each sampling site, by conducting a simple linear mixing model between river runoff and seawater. Here we apply a simple linear regression to assess the change in salinity as a function of ^87^Sr/^86^Sr variability. In order to do so, we use the global isotopic curve as the seawater ^87^Sr/^86^Sr endmember^23^, which is assumed to correspond to an open marine salinity value of 35. The strontium isotopic composition of the mineral waters described above^19^ represent the riverine endmember with a salinity of 0 (Supplementary Data 2). Using the maximum mineral water ^87^Sr/^86^Sr value for each basin results in a minimal estimate for salinity change and vice versa, since these short-term variations are influenced by both the rate of runoff and the strontium composition of the fresh water influx. We use the maximal range of ^87^Sr/^86^Sr within each shell to assess average perennial variability in salinity over the life time of the bivalves.

**Fig. 3:**
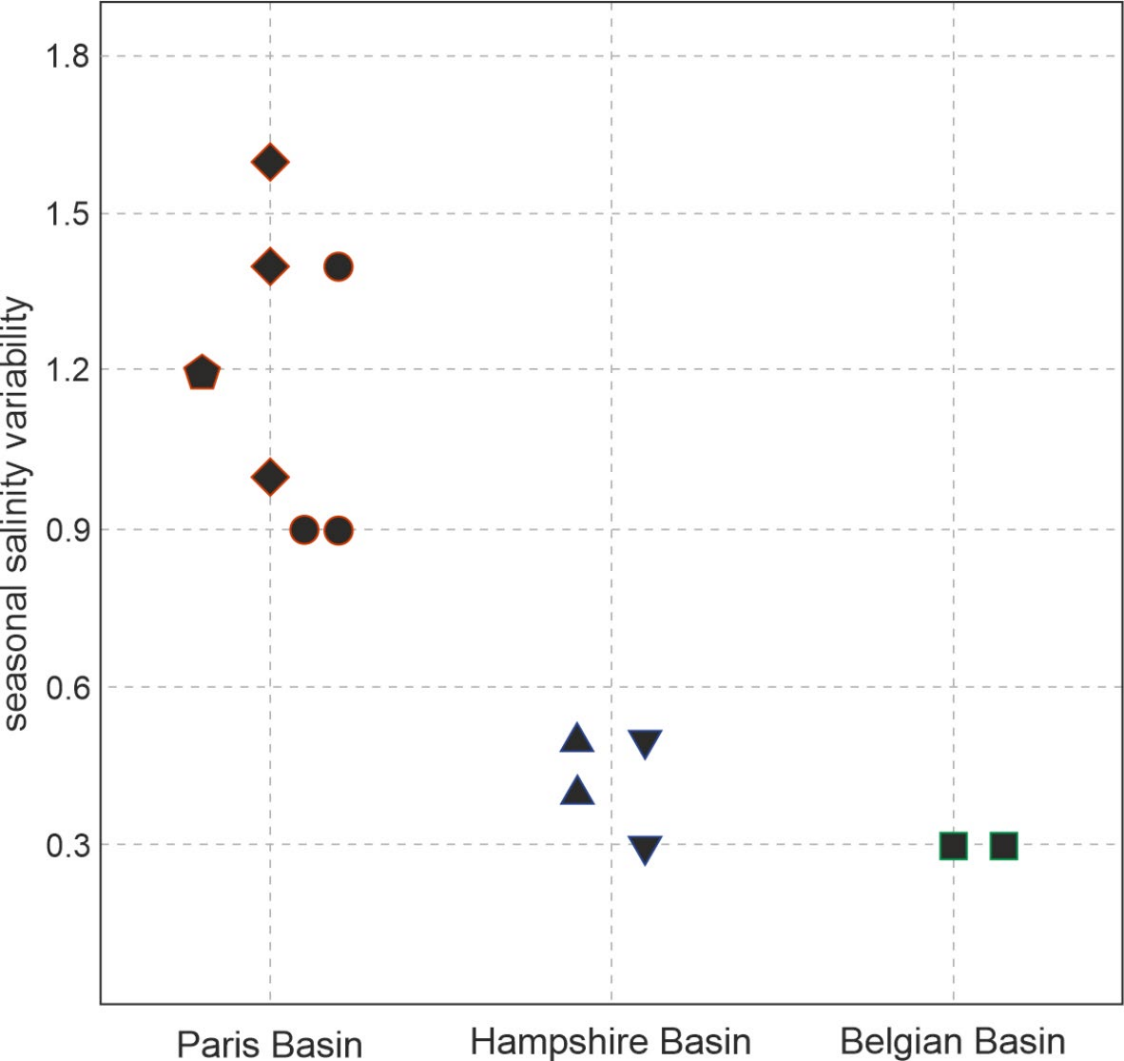
Mean perennial salinity variability. Mean salinity variations calculated for each individual shell using the maximum range of ^87^Sr/^86^Sr of each specimen (Fig. 2b) and the mean ^87^Sr/^86^Sr for the mineral waters^19^ of each of the three basins (Supplementary Data 2). Symbol and colour coding are the same as in Fig. 2.

Following this approach, we find that the relative change in salinity for the paleo North Sea basin to range between 0.2 and 4.6 on the practical scale with no systematic long-term trend. However, a spatial difference between the three basins in the relative change in salinity driven by seasonal fresh water input can be observed (Fig. 3). In terms of intra-site/specimen variability, the shells from the Belgian Basin show the lowest degree, with reconstructed salinity 0.2 to 1.5 units below that of normal seawater. In this case, the comparatively small degree of intra-shell ^87^Sr/^86^Sr heterogeneity of ~ 77 × 10^−6^ in comparison to the large offset from the seawater composition reflects a riverine input with a low rate of runoff, but a more radiogenic strontium isotopic composition. In the case of the Hampshire Basin, the range of freshening estimates are greater than those from the Belgian Basin, from ~ 0.2 up to 2.8 units below normal seawater. These findings agree well with the previously discussed paleo-geographical and oceanographic conditions in the Hampshire Basin, which was determined by similar background geology ^87^Sr/^86^Sr values as in the Belgian Basin but therefore requiring a considerably greater degree of fluvial discharge at this site. The specimens from the Paris Basin reveal even higher salinity variations, ranging from 0.4 to 2.6 units below normal seawater in Fleury-La-Rivière to a 4.6 unit freshening in Chaumont-en-Vexin, again mirroring the hydrologically different conditions between the eastern and western part of the basin. Therefore, the observed differences in ^87^Sr/^86^Sr between the three investigated sampling sites cannot be attributed to the background geology alone, but must also be influenced by different amounts of freshwater input.

Given the absence, to our knowledge, of a data set with comparably high temporal resolution, whether for fossil or recent bivalves, it is reasonable to question whether the observed short-term fluctuations in strontium isotopic composition might be influenced by factors other than seasonal variable fluvial discharge. However, the implied hydrological conditions are in good agreement with previous reconstructions of the Eocene Paleo North Sea (e.g. stable oxygen and clumped isotopes), revealing a heterogeneously distributed fresh water input between the different adjacent basins, geological periods and seasons^39–41^. Kniest et al.^42^ used seasonally resolved dual clumped isotopes (Δ_47_ + Δ_48_) to reconstruct sub-annual changes in salinity at Le Guépelle, analysing the same bivalve shell as presented in this study. The reconstructed δ^18^O_SW_ revealed an enhanced summer runoff and shifts in salinity of 2.5 to 3.0 units, which agrees well with the freshening-driven decrease in salinity derived from the ^87^Sr/^86^Sr data of this study. However, local to regional conditions of the hydrological system appear to be well recorded by the radiogenic strontium composition, large changes in the climate system are not visible in the data set, i.e. as general trends due to global cooling during the early to middle Eocene^2^.

### Ba/Ca baseline as an additional freshwater tracer in Eocene bivalves

In bivalves, Ba/Ca profiles along the growth direction of the shell typically consists of a flat and almost invariant baseline with occasionally large and relatively sharp peaks, which can exceed the background by up to several orders of magnitude^9,43–46^. This is also the case for all specimens examined in this study. While the cause of these peaks is still debated^9,43,45–47^, the Ba/Ca baseline is well correlated to the Ba/Ca ratio of the seawater in which the organism lived^9,43,48^. Similar to the strontium, the major source of barium to seawater in marginal environments is via the input of terrestrial material by fluvial discharge and depends on the chemical composition of the hinterland geology and the rate of runoff^10^.

However, we observe considerably less Ba/Ca_BL_ variability between and among the basins compared to our strontium isotope results. In general, baseline values fall within an interval of 10 to 20 µmol/mol, independent of sample site. The lowest Ba/Ca_BL_ in the samples from the Belgian Basin with an average value of 12.4 µmol/mol, implying a lower rate of runoff compared to our estimate based on the strontium isotopic composition of the same shells. In the Paris Basin the geographical difference in the rate of runoff revealed by the locally different ^87^Sr/^86^Sr values is not reflected by the barium baselines of the shells. The mean values of Ba/Ca_BL_ between the western and eastern sampling sites are almost identical with 16.9 µmol/mol and 17.4 µmol/mol in Chaumont-en-Vexin and Fleury-la-Rivière, respectively. The specimen from Le Guépelle exhibits a slightly lower value of 13.9 µmol/mol. An exception to this overall discordant Ba/Ca and ^87^Sr/^86^Sr results are the specimens from the Hampshire Basin, which are characterised by substantially higher values of 16.8 µmol/mol to 25.0 µmol/mol on average from the early to the middle Eocene, similar to the increasing radiogenic ^87^Sr/^86^Sr values and suggesting a higher riverine input.

The low spatial variability of Ba/Ca_BL_ suggest that the soluble barium concentration in the background geology of the southern Paleo North Sea appears to have been less variable than the strontium isotopic composition. This is perhaps unsurprising on a regional scale, although we note that modern global rivers are characterised by very different [Ba]^8^. However, due to the relatively short residence time of barium in seawater of about 10 ka^49^, the identification of spatiotemporal deviations in Ba/Ca ratio from global seawater is more hallenging than for strontium isotopes. In this context, the low temporal variations along the examined 13 Ma time interval are rather unexpected, indicating a more constant Ba/Ca ratio of the seawater than implied by the residence time.

In addition, we note that comparing the barium baseline of the fossil shells presented here with values derived from recent bivalves (generally < 4 µmol/mol^9,43–46^) demonstrate that, Eocene Ba/Ca_BL_ is about three to ten times higher. While a higher species-specific apparent distribution coefficient (D_Ba_) could result in a higher degree of barium incorporation into the shell, modern bivalves are characterised by only minor differences in D_Ba_ between species and shell mineralogies^9,43,50^. Modern surface waters are typically depleted in Ba compared to the deep sea and are therefore undersaturated with respect to barite (BaSO_4_)^51,52^. Given an Eocene Ba concentation an order of magnitude higher, as inferred by the bivalve Ba/Ca_BL_ values reported here, would - all else being equal - likely result in seawater oversaturated with respect to barite, resulting in precipitation, which should remove Ba from the seawater and limit its bioavailability. However, seawater major-ion chemistry, in particular Ca^2+^, Mg^2+^ or SO_4_^2-^, have changed considerably during the Cenozoic. The Eocene Ca concentration was about twice as high as in modern oceans, while Mg and SO_4_^2-^ concentrations have almost doubled over the last 40 Ma^53,54^. These differences in seawater composition would have acted to shift barite saturation of the average ocean in the opposite direction, thereby allowing a higher seawater Ba concentration and thus greater-than-modern bivalve Ba/Ca.

## Conclusion

In this study the barium concentration and isotopic composition of strontium of Eocene bivalves was used to reconstruct river runoff in three adjacent basins of the paleo North Sea. Laser ablation (multi-collector) inductively-coupled-plasma mass-spectrometry facilitated high resolution measurements on thirteen specimens from different geological stages, allowing a reconstruction along different spatiotemporal resolutions. The ^87^Sr/^86^Sr values of the analysed shells constrain pronounced riverine input with varying rates of runoff within and among the examined marginal sea basins. At the regional scale, deviations in the strontium isotopic composition of the shells from the global seawater record, accounting for spatial and temporal variability in the likely composition of Eocene river water, also reflects the oceanographic evolution of the different basins^18,20,34^. In addition, leveraging the spatially-resolved nature of our strontium isotope measurements enables us to demonstrate that short-term (seasonal scale) salinity fluctuations derived from the observed range in ^87^Sr/^86^Sr variability over perennial intervals agrees well with estimates based on other proxy systems, such as oxygen or clumped isotopes^42^. In contrast, we find that comparative barium baseline data (Ba/Ca_BL_) exhibit only minor changes during the considered time period, being apparently less sensitive to hydrological changes despite its demonstrable utility as a salinity proxy in certain settings in the recent geological past^10^.

Using laser ablation allows the fast and precise measurement of radiogenic strontium isotopes in carbonate shells at a high spatial, and therefore temporal, resolution. This has enabled us to achieve a previously unavailable spatiotemporal characterisation of past riverine discharge systems, describing them at a resolution that is considerably higher than the typical output of climate model simulations^42^. We therefore advocate for the application of this proxy system for the reconstruction of paleo environments, especially in ocean settings likely to be characterised by substantial influx of fresh water. In particular, we demonstrate that while the strontium isotopic composition as measured in the bivalve shells is a function of both freshwater-derived salinity change and background geology, it can nonetheless be utilized to detect hydrological patterns in regions where the paleo strontium isotopic composition of river water can be reconstructed with a good degree of confidence. Therefore, the proxy may serve as a valuable addition to more time and material intensive techniques, such as oxygen or clumped isotopes, allowing more differentiated identification of hydrological systems for the prediction of future climate scenarios.

## Materials and methods

### Sample material

Fossil bivalve shells of the species *Venericor planicosta* (Lamarck), *Venericor planicosta lerichei* (Glibert & Van de Poel), *Venericor* sp. nov. and *Crassatella ponderosa* (Gmelin) were used as archives. All taxa produced aragonitic shells, lived as shallowly burrowing suspension feeders and were widespread in the southern paleo North Sea during the Eocene. The shells originate from three geological sites within the Paris Basin (Chaumont-en-Vexin, Fleury-La-Rivière, Le Guépelle), two locations in the Hampshire Basin (Studley Wood, Nursling) and one location within the Belgian basin (Aalter), representing a time span of ~ 13 Ma from the Middle Ypresian (53 Ma) to the early Bartonian (40 Ma)^16,55–57,35,58–60^. All examined locations represent shallow marine environments, which are dominated by fine sand sedimentation with intermitted calcareous beds in the Paris and Belgian Basin and recurring clay-rich layers in the Hampshire Basin^55–57,38,58,60^.

### Sample preparation and preservation screening

For each specimen, polished 150 μm-thick sections of the plane of maximum growth were produced. Analyses were conducted on the hinge plate (Supplementary Fig. S1a), due to the compactness and geometry of increments in this shell area, which means that this region of the shell is likely to be more resistant to diagenetic alteration and provides clear growth lines, such that spatially resolved analyses can be readily aligned perpendicular to the of shell growth pattern^45^.

To assess aragonitic preservation and to identify potential recrystallization of the shell material, the crystal structure of each specimen was determined by Raman spectroscopy. Raman analyses were performed using a WITec alpha 300R confocal micro-Raman microscope, located at the Institute of Geoscience at Goethe University in Frankfurt a.M. For the measurements the excitation laser (532 nm) was operated at 20–40 mW with an integration time of 0.2 s, 10 total accumulations and a holographic grating of 600 grooves mm^−1^. The resulting Raman spectra indicate aragonitic preservation in the case of all sampled shells (Supplementary Fig. S1d).

### Elemental and isotopic analysis

Measurements of Ba/Ca and ^87^Sr/^86^Sr were conducted using laser ablation (MC)-ICPMS at the Frankfurt Isotope and Element Research Center (FIERCE), using a RESOlution S-155 193 nm ArF excimer laser system (formerly Resonetics LLC, now Applied Spectra Inc) coupled to either a single-collector (Ba/Ca) or multi-collector ICPMS (^87^Sr/^86^Sr).

For the analysis of Ba/Ca ratios the laser system was coupled to an Element XR sector field ICP-MS (Thermo Fisher Scientific) tuned to ensure robust plasma conditions (Th/U = 1 ± 0.1, ThO^+^/Th^+^ < 0.5%, m/z 22/44 < 2%) while simultaneously maximising sensitivity (> 6 M cps ^238^U, NIST SRM612, 60 μm beam diameter, 6 Hz, ~ 6 J/cm^2^). Measurements were conducted in line scan mode with a beam diameter of 50 μm and a scan speed of 10 μm/s with a pulse rate of 10 Hz. All laser tracks were pre-ablated to remove possible surface contamination using a laser beam diameter, scan speed and pulse rate of 75 μm, 20 μm/s and 20 Hz, respectively. Samples and standard materials were analysed in an identical manner.

NIST SRM 612 was employed as external standard for the calibration. Monitored masses included ^43^Ca and ^138^Ba, with a NIST SRM612 Ba concentration of 39.7 µg/g used for calibration^61^. ^43^Ca was measured as internal standard and used for Ba/Ca calculation. The natural carbonate standards JCp-1-NP and JCt-1-NP and the synthetic USGS standard MACS-3-NP were used as external standards and repeatedly measured to assess accuracy and precision of the analyses. The results of the standard measurements are reported as averages across the three analytical sessions in which the samples were analysed. The measured Ba/Ca ratios for the three reference materials are well within reported values, yielding 7.8 ± 0.4 µmol/mol for JCp-1-NP (reference value = 7.1 ± 0.8 µmol/mol, accuracy: 9.86%), 5.0 ± 0.7 µmol/mol for JCt-1-NP (reference value = 4.1 ± 0.5 µmol/mol, accuracy: 21.95%) and 43.6 ± 2.0 µmol/mol for MACS3-NP (reference value = 46.2 ± 0.9 mmol/mol, accuracy: -5.63%)^62,63^.

Spatially resolved strontium isotope measurements were conducted using a Neptune Plus multi-collector ICP-MS (Thermo Fisher Scientific) connected to the same laser ablation system described above. The instrument was optimised for maximum sensitivity while ensuring the oxide formation rate (^238^U^16^O/^238^U) remained below 0.8%. Data were acquired in static mode using 10^11^ Ω amplifier for masses 83, 84, 85, 86, 87 and 88 on Faraday cups L4, L2, L1, C, H2, H3 and 10^13^ Ω amplifiers for masses 83.5 and 86.5 (L3, H1), which altogether allowed interferences from Kr and Rb, doubly-charged ions of Yb and Er, as well as any Ca dimers or argides to be monitored. All data were corrected using on-peak background measurements. Sample ablation was executed with 60 μm diameter quadratic laser spots, a fluence of 2.5 J/cm^2^ and a repetition rate of 8 Hz for 36.4 s. The laser spots were placed parallel to the Ba/Ca tracks with a spacing of 350 μm between the spots (Supplementary Fig. S1a). Instrumental mass bias was corrected using the exponential law and the natural ^86^Sr/^88^Sr ratio of 0.1194^64,65^. Correction for the Kr interference on the masses 84 and 86, induced by impurities in the Ar plasma gas, was achieved by gas blank subtraction. The isobaric interference produced by Rb^+^ or Er^2+^ and Yb^2+^ were corrected applying empirical offset factors, which were determined from analyses of NIST SRM 612. Background, interference, and mass bias corrected ^84^Sr/^86^Sr and ^87^Sr/^86^Sr ratios outliers were removed by applying a 2SD criterion to all intensities with a ^88^Sr signal > 0.5 V. Following mass bias and interference correction, measurements yield the accepted natural ^84^Sr/^86^Sr of 0.05649 ± 0.00009 (2 SE). In order to assess the accuracy and precision of the method described above, the in-house standards MIR-A (Plagioclase megacryst, 3000 µg/g) and a recent oyster shell (*Crassostrea gigas*, Thunberg) from the Jade Bight (Germany) were measured repeatedly during the analysis in the same manner as the samples. On average, the measurements of MIR-A yield an ^87^Sr/^86^Sr value of 0.703082 ± 0.000037 (2SD), which is in good agreement with previously reported results from TIMS measurements of 0.703096 ± 0.000076 (2SD)^66^. The oyster shell yielded a mean ^87^Sr/^86^Sr of 0.709206 ± 0.000031 (2SD), which fits well with the mean modern seawater value of 0.709172 ± 0.000021 (2SD)^6^. Furthermore, the oyster shell value matches well with previously observed ^87^Sr/^86^Sr values for *C. gigas* from the southern North Sea and the English Channel, revealing average values ranging from 0.70917 to 0.70923^6^ (supplementary data 4).

### Ba/Ca baseline determination

The statistical distributions of measured Ba/Ca values are strongly right skewed for all shell records (Supplementary Fig. S1c), due to a dominant low Ba/Ca baseline punctuated by sharp peaks with Ba/Ca values 10 to 50 higher than the background. Here, we use the mode of the probability density function as a threshold to distinguish between background and peaks. Baseline values (Ba/Ca_BL_) and its variability are then defined as the mean and 2SD variability of all values smaller than the mode of all measurements within a single shell.

## Electronic supplementary material

Below is the link to the electronic supplementary material.

Supplementary Material

Supplementary data 1-4

Supplementary data 5

## Data Availability

The datasets analysed during the current study are available in the Zenodo repository (zenodo.org/records/14178112).
