# Supplementary material for: Spatiotemporal changes in riverine input into the Eocene North Sea revealed by strontium isotope and barium analysis of bivalve shells
Source: Sci Rep. 2024 Nov 20;14:28806. doi: 10.1038/s41598-024-79779-0 (PMC11579471; doi:10.1038/s41598-024-79779-0)
Supplement: Supplementary file 1 — Supplementary Material [file 41598_2024_79779_MOESM1_ESM.pdf]

Supplementary Information accompanying the manuscript titled:

"Spatiotemporal changes in riverine input into the Eocene North Sea revealed by strontium isotope and barium analysis of bivalve shells"

Jorit F. Kniest[1,2,9*], David Evans[1,2,3], Axel Gerdes[1,2], Marjorie Cantine[1,2,4], Jonathan A. Todd[5], Julia D. Sigwart[6], Johan Vellekoop[7,8], Wolfgang Müller[1,2], Silke Voigt[1,2], Jacek Raddatz[1,2,9]

1 Institute of Geosciences, Goethe University Frankfurt, Frankfurt am Main, Germany
2 Frankfurt Isotope and Element Research Center (FIERCE), Goethe University Frankfurt, Frankfurt am Main, Germany
3 School of Ocean & Earth Science, University of Southampton, Southampton, UK
4 Department of Earth and Space Sciences, University of Washington, Seattle, USA
5 The Natural History Museum, London, UK
6 Department of Marine Zoology, Senckenberg Institute and Natural History Museum, Frankfurt am Main, Germany
7 Department of Earth and Environmental Sciences, KU Leuven, Leuven, Belgium
8 Operational Directorate Earth and History of Life, Institute of Natural Sciences, Brussels, Belgium
9 GEOMAR Helmholtz Centre for Ocean Research Kiel, Kiel, Germany
* Corresponding Author (jkniest@geomar.de)

Overview of Supplementary Data files

- Supplementary material: supplementary Figure S1
- Supplementary data 1: Element and isotope data for each individual shell
- Supplementary data 2: $^{87}Sr/^{86}Sr$ salinity variability reconstruction
- Supplementary data 3: Stratigraphy for sampling locations
- Supplementary data 4: $^{87}Sr/^{86}Sr$ data for recent oyster shells
- Supplementary data 5: Extended strontium isotope data

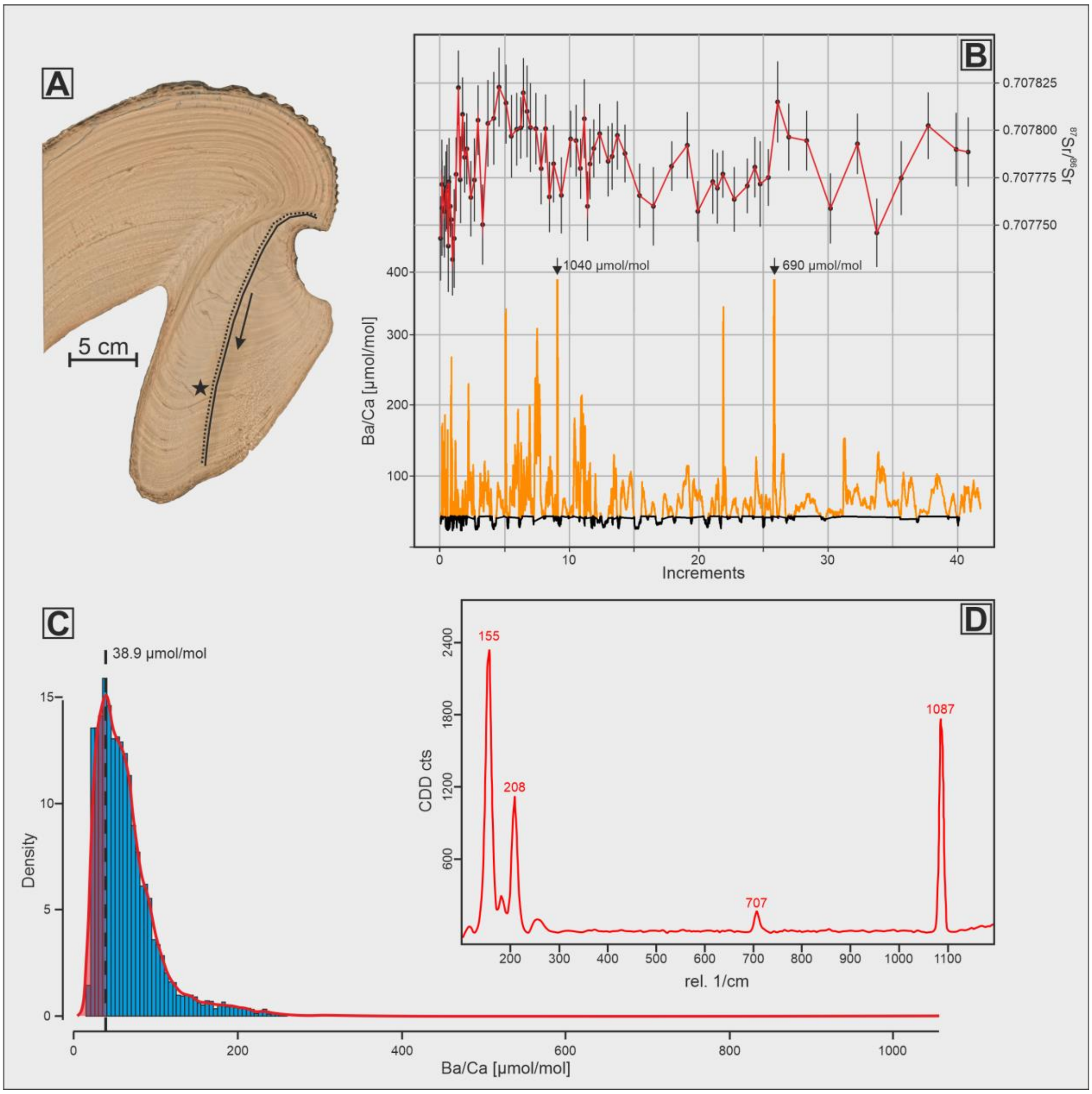

Figure S1 – **Example overview of measurements and resulting data along a bivalve shell** – The cross section and geochemical data belong to the specimen "VP_SW2" (Studley Wood, *Venericor planicosta*); all specimens used in this study were analysed in the same manner. A: cross section of the hinge plate with the positions of the laser pathway used to measure Ba/Ca (black line) and laser spots used to measure $^{87}Sr/^{86}Sr$ (black squares). The black arrow

indicates that the laser ablation pathways was parallel to growth direction, the black star shows the position of the Raman measurement close to the laser track; B: Radiogenic strontium isotope and barium to calcium ratios plotted against shell growth increments, counted relative to the hinge plate. $^{87}Sr/^{86}Sr$ is displayed with 2SE uncertainties (black vertical lines) for each individual laser spot. Ba/Ca data is shown in orange. Two Ba/Ca peaks extend above the y-axis limit and are labelled by black arrows with corresponding peak values. The black portion of the Ba/Ca data indicates values below the barium baseline (Ba/Ca_{BL}); C: Histogram and density probability function of Ba/Ca measurements. The mode (dashed line) marks the upper limit of the Ba/Ca baseline. Values smaller than the mode (red area) are used to calculate the mean and standard deviation of Ba/Ca_{BL} (s. Figure 2); D: Raman spectrum showing the characteristic features of aragonite with (e.g.) the distinct lattice mode vibrations at 155 and 208 cm^{-1}.
